# Production, characterization and techno-economic evaluation of *Aspergillus* fusant l-asparaginase

**DOI:** 10.1186/s13568-022-01505-8

**Published:** 2023-01-06

**Authors:** Atim Asitok, Maurice Ekpenyong, Andrew Amenaghawon, Ernest Akwagiobe, Marcus Asuquo, Anitha Rao, David Ubi, Juliet Iheanacho, Joyce Etiosa, Agnes Antai, Joseph Essien, Sylvester Antai

**Affiliations:** 1 Environmental Microbiology and Biotechnology Unit, Department of Microbiology, University of Calabar, Calabar, Nigeria; 2 University of Calabar Collection of Microorganisms (UCCM), Department of Microbiology, University of Calabar, Calabar, Nigeria; 3 Department of Chemical Engineering, University of Benin, Benin City, Nigeria; 4 Industrial Microbiology and Biotechnology Unit, Department of Microbiology, Faculty of Biological Sciences, University of Calabar, Calabar, Nigeria; 5 Department of Hematology, University of Calabar Teaching Hospital, Calabar, Nigeria; 6 Department of Economics, Faculty of Social Sciences, University of Calabar, Calabar, Nigeria; 7 Department of Microbiology, Faculty of Science, University of Uyo, Uyo, Nigeria

**Keywords:** Protoplast fusion, l-ASNase, Sensitivity analysis, Anti-leukemic activity, Acrylamide mitigation, Techno-economics

## Abstract

Protoplast fusion is one of the most reliable methods of introducing desirable traits into industrially-promising fungal strains. It harnesses the entire genomic repertoire of fusing microorganisms by routing the natural barrier and genetic incompatibility between them. In the present study, the axenic culture of a thermo-halotolerant strain of *Aspergillus candidus* (Asp-C) produced an anti-leukemic l-asparaginase (l-ASNase) while a xylan-degrading strain of *Aspergillus sydowii* (Asp-S) produced the acrylamide-reduction type. Protoplast fusion of the wild strains generated Fusant-06 with improved anti-leukemic and acrylamide reduction potentials. Submerged fed-batch fermentation was preferred to batch and continuous modes on the basis of impressive techno-economics. Fusant-06 l-ASNase was purified by PEG/Na^+^ citrate aqueous two-phase system (ATPS) to 146.21-fold and global sensitivity analysis report revealed polymer molecular weight and citrate concentration as major determinants of yield and purification factor, respectively. The enzyme was characterized by molecular weight, amino acid profile, activity and stability to chemical agents. Michaelis–Menten kinetics, evaluated under optimum conditions gave *K*_*m*_, *V*_*ma*x_, *K*_*cat*_*,* and *K*_*cat*_*/K*_*m*_ as 6.67 × 10^–5^ M, 1666.67 µmolmin^−1^ mg^−1^ protein, 3.88 × 10^4^ min^−1^ and 5.81 × 10^8^ M^−1^.min^−1^ respectively. In-vitro cytotoxicity of HL-60 cell lines by Fusant-06 l-ASNase improved significantly from their respective wild strains. Stability of Fusant-06 l-ASNase over a wide range of pH, temperature and NaCl concentration, coupled with its micromolar *K*_*m*_ value, confers commercial and therapeutic value on the product. Free-radical scavenging and acrylamide reduction activities were intermediate and the conferred thermo-halo-stability could be exploited for sustainable clinical and food industry applications.

**Supplementary Information:**

The online version contains supplementary material available at 10.1186/s13568-022-01505-8.

## Key points

- Sensitivity analysis confirmed optimization conditions for ATPS L-ASNase recovery
- Fed-batch fermentation outperformed other modes in techno-economic evaluations
- Fusant improved clinical and industrial characteristics indicate recombination success

**Supplementary Information:**

The online version contains supplementary material available at 10.1186/s13568-022-01505-8.

## Introduction

l-asparaginases (l-asparagine amidohydrolase, EC 3.5.1.1) catalyze the hydrolysis of L-asparagine into l-aspartic acid and ammonia. They cause intracellular depletion of serum l-asparagine leading to general impairment in glutamine metabolism through the tricarboxylic acid cycle, thus interfering with the ability of lymphoblasts to take up the amino acid (Vala et al. 2018). Additionally, they are involved in the prevention of other kinds of cancers through scavenging of free-radicals and acrylamide reduction in fried, roasted or baked foods (Shakambari et al. 2017). They are produced naturally by all biological systems, typically plants and microorganisms, and could be expressed in biotechnologically-safe heterologous hosts (Mihooliya et al. 2020). A study comparing plant- and bacterial-derived l-ASNase revealed superiority of plant l-ASNases over bacterial counterparts in terms of toxicity (Al-Hazmi and Naguib 2022). The untoward reactions that accompany treatment with prokaryotic l-ASNases appear to not exist for plant l-ASNases which are rather clinically innocuous. Bacterial l-ASNases show immuno-toxicities and allergenic reactions derived, in large part, from cross-reactivity with l-glutamine which has been linked to the huge genetic difference between prokaryotic and eukaryotic classifications of life (Cachumba et al. 2016). Besides, glutamine is the dominant nitrogen source in the blood and its secondary hydrolysis by l-ASNase naturally results in a myriad of untoward reactions.

One of the trendy attempts to circumvent the toxicity problems of prokaryotic l-ASNases includes bio-prospecting eukaryotic micro-bioresource for l-ASNase production as they present little-to-no glutaminase activity (Ekpenyong et al. 2021a). Their low glutaminase activity has been reported to prolong pre-clinical activity against acute lymphoblastic leukemia (Chan et al. 2019). Accordingly, a number of yeast genera have been reported with significant l-ASNase-producing abilities mainly from the genera *Candida*, *Yarrowia* and *Rhodotorula* while *Aspergillus*, *Penicillium*, and *Fusarium* dominate literature for l-ASNase-producing mold genera (Lopes et al. 2017).

A common huddle with fermentative production of L-ASNases and other microbially-derived value-added products is low yield because microorganisms maintain tight control over biosynthesis of metabolites, especially extracellularly-secreted ones (Sadh et al. 2018; Pinu et al. 2018). A combined use of hyper-producing strains obtained naturally or through mutagenesis, protoplast fusion and genetic engineering, especially with CRISPR/Cas9 system (Zou et al. 2019; Zhang et al. 2021; Hu et al. 2021), and design of experiments by response surface methodology and artificial neural network, has significantly improved yield (Farjaminezhad and Garoosi 2021; Asitok et al. 2022a; Kusuma et al. 2022).

Protoplast fusion is a method of strain improvement employed in modern biotechnology to recombine genomes with desirable traits in producer organisms to obtain superior strains for biotechnological applications (Strom and Bushley 2016). Intraspecific (Klinsupa et al. 2016), interspecific (Zhu et al. 2010) and intergeneric (El-Gendy et al. 2017) protoplast fusion techniques have all been reported for improved production of pigment, β-glucosidase and l-ASNase, respectively. The main thrust of protoplast fusion is to harness the entire genomic repertoire of the fusing microorganisms by routing the natural barrier and genetic incompatibility between them. However, l-asparaginase enhancement through interspecific mold protoplast fusion has not been reported till date.

Design of experiments for optimization of nutritional, environmental and bioprocess operational conditions by response surface methodology—a statistical approach that is limited to addressing non-noisy quadratic functions—has been copiously reported (Farjaminezhad and Garoosi 2021; Asitok et al. 2022c). However, a significant part of process variations owing to input–output interactions is non-linear and stochastic, and may require neural network approaches for elucidation (Ekpenyong et al. 2021a; Asitok et al. 2022d). To determine which of the many input factors contributed significantly to the response and by how much, a sensitivity analysis is frequently conducted often using the global sensitivity index analysis (Amenaghawon et al. 2022).

The present study reports successful improvement of l-ASNase production through interspecific protoplast fusion of *Aspergillus candidus* strain UCCM 00117 and *Aspergillus sydowii* strain UCCM 00124 and sensitivity analysis of the response surface conditions for its purification. The enzyme demonstrated enhanced anti-leukemic, acrylamide reduction and anti-oxidation potentials, with significantly improved physicochemical characteristics which were lacking in wild strains. Improvement of l-ASNase production by interspecific mold protoplast fusion is here reported for the first time.

## Materials and methods

### Study mold strains

Wild-type strains of *Aspergillus candidus* UCCM 00117 (Asp-C) and *Aspergillus sydowii* UCCM 00124 (Asp-S) were obtained from University of Calabar Collection of Microorganisms (UCCM) (www.wfcc.info/ccinfo/collection/by_id/652) and reactivated in Czapek-Dox agar (CDA) medium (Sigma-Aldrich, USA) for 96 h at 30 ºC. The strains were grown in minimal media containing 1% (w/v) xylan at 30 ºC and on PDA-NaCl medium containing 25% (w/v) NaCl at 55 ºC. The minimal medium contained 0.35% NaNO_2_, 0.15% K_2_HPO_4_, 0.05% MgSO4.7H_2_O, 0.05% KCl, 0.001% FeSO_4_.7H_2_O supplemented with 1% (w/v) xylan (Sigma Aldrich, Germany) (Brandt et al. 2020). Xylanase production with no-growth on PDA-25% NaCl medium was characteristic of Asp-S while non-xylanase production but luxuriant growth on PDA-25% NaCl was characteristic of Asp-C.

### Preparation of protoplasts from wild Aspergillus strains

Effects of mycelial age (36, 72 and 108 h), pH (4, 6 and 8) and incubation time (2, 4 and 6 h) on protoplast isolation were investigated using Box-Benkhen design (BBD) of a response surface methodology (RSM) in Design Expert version 12 (StatEase, Minnesota, USA) (Farjaminezhad and Garoosi 2021). The optimized factor levels were used for protoplast isolation in a medium composed (g.L^−1^) of glucose 1; KH_2_PO_4_ 0.1; KCl 0.5; MgSO_4_.7H_2_O 0.05; casamino acid 0.1, at optimized pH. Flasks were inoculated, upon cooling, with 1 mL of 3.5 × 10^6^ sfu.mL^−1^ of each strain and incubated, with agitation, for an optimized period of time (h). Germlings of optimized age were harvested by centrifugation at 2655.25 × *g* for 20 min and washed twice in 5 mL 25 mM Tris–HCl buffer at pH 7.5. Pellets were re-suspended in a buffer mixture (5 mL) of 25 mM each of Tris–HCl at pH 7.5 and CaCl_2_, and 1.2 mM sorbitol (Klinsupa et al. 2016). Thereafter, the obtained suspensions were suspended in lytic enzyme mixture comprising glucanase (1200 U), cellulase (850 U), protease (875 U) and chitinase (180 U) and incubated for an optimized period at 30 ºC with slow shaking (50 rpm). Protoplasts were separated from mycelia using sintered glass filter and washed again in the osmotically-stabilized buffer mixture to rid the preparation of lytic enzymes.

### Protoplast fusion

Protoplast suspensions of both strains (1.5 mL, 8.5 × 10^6^ cells.mL^−1^) were centrifuged at 663.81 × *g* for 15 min. Pellets were re-suspended in 1 mL 40% (w.v^−1^) 6000 kDa polyethylene glycol in 10 mM CaCl_2_ mixed with 0.05 M glycine at pH 7.5, thereafter incubated at 30 ºC for 30 min. Suspension was diluted in 6 mL 0.8 M sorbitol-containing MM before centrifuging at 2150.75 × *g* for 15 min. Pellets of fusants were washed twice in sorbitol solution (8 mL of 0.8 M) and re-suspended in 5 mL of same solution.

Protoplasts were regenerated on yeast malt potato dextrose agar (YMPDA) with 0.8 M of sorbitol or sucrose as osmotic agents. Fifty microliters (50 µL) of protoplast suspension (3.5 × 10^3^ sfu.mL^−1^) was plated on the surface of sterile regenerating medium containing osmotic stabilizers. Plates were incubated for 72–96 h at 30 ºC. Regenerated colonies were characterized by macro-morphology on CDA for differences in mycelial growth pattern and by l-ASNase activity, combined potentials of xylanase production at 30 ºC and growth on PDA-25% NaCl at 55 ºC. Fusants with prospects were preserved for further studies on CDA plates in a refrigerator and the most promising was maintained in sterile soil and deposited at the UCCM and assigned the collection number UCCM 00130F06.

### Comparative fermentative production of L-ASNase by protoplast fusant and cost analysis

l-ASNase production by submerged and solid-state fermentation types were compared in shake flasks. Submerged fermentation (SmF) medium was as detailed in Ekpenyong et al. (2021a). Solid-state fermentation (SSF) utilized sugarcane bagasse (2 g dry mass) as support material in 100 mL Erlenmeyer flasks and 10 mL solution mixture of 10% test spore suspension and 90% of fermentation medium with same composition as SmF. Inoculated flasks for SmF were incubated at 30ºC on orbital shaker (100 rpm) for 96 h while SSF flasks were incubated in a chamber at 30 ºC and 80% humidity (Cachumba et al. 2019). The better fermentation type, assessed by l-ASNase activity, was then operated by batch, fed-batch or continuous mode in 5 L bioreactors (BioStat^(R)^ CPlus, Sartorius Stedim Biotech, Germany) with 3.5 L working volume for l-ASNase production by the fusant.

In the batch mode, the optimized medium reported in Ekpenyong et al. (2021a) was used. Sterilization was conducted in situ and inoculation with 3% (v.v^−1^) spore suspension. The bioreactor was operated as described in Asitok et al. (2022a) and determinations of total protein (Bradford 1976), biomass concentration (Rodrigues et al. 2006), l-ASNase activity (Imada et al. 1973) and residual carbohydrate (Miller 1959) were made at 6 h interval for 96 h. Briefly, l-ASNase assay protocol by Imada et al. (1973) involved dissolution of 0.04 M of l-asparagine (0.5 mL) in 0.5 M (0.5 mL) Tris–HCl buffer (pH 7.2) and adding the mixture to 0.5 mL of cell-free fermentation broth of fusant mold and making up total reaction volume to 2 mL with sterile distilled water. The preparation was incubated in a water bath at 37 ºC for 30 min and reaction stopped by adding 0.5 mL of 1.5 M trichloroacetic acid. One unit of enzyme activity was defined as the amount of enzyme that liberated one micromole of ammonia from substrate in 1 min. Means of triplicate determinations were subjected to Pearson’s bivariate correlation and subsequently employed for logistic and/or modified Gompertz model fitting (Ekpenyong et al. 2021b).

In the fed-batch mode, fermentation started batch-wise for 24 h with same medium used during batch mode except that the starting molasses concentration was reduced to 30 g.L^−1^. Medium pH was adjusted to 5.8, temperature to 50ºC, dissolved oxygen to 45% using the agitation cascade of the bioreactor at 100–600 rpm and 1.5 vvm aeration. Feeding solution was composed of 50 g.L^−1^ molasses, 44 g.L^−1^ asparagine and 0.5 g.L^−1^ MnCl_2_ using a peristaltic pump. Feed rate of the solution was calculated using equation below;

1 $$F=\frac{\mu {X}_{0}{V}_{0}{e}^{\mu t}}{{Y}_{X/S}{S}_{0}}$$

where *F* (h^−1^) is feed rate, *X*_0_ (g.L^−1^) is mold biomass concentration at end of batch operation, *V*_0_ is volume (L) of spent medium at the end of batch operation and *S*_0_ (g.L^−1^) is total substrate concentration, *µ* (h^−1^) is the specific growth rate, *X/S* (g.g^−1^) is biomass yield on substrate determined from the batch part of the fed-batch fermentation using Eqs. 2 and 3 below;

2 $$\mu =\frac{1}{X}\frac{dx}{dt}$$

3 $${Y}_{X/S}=\frac{{X}_{m}-{X}_{0}}{{S}_{0}-{S}_{m}}$$

where *X*_m_ is maximum fungal dry cell weight (DCW) (g.L^−1^) at time *t*, *X*_0_ is DCW (g.L^−1^) at *t* = 0, *S*_0_ is initial substrate concentration (g.L^−1^) at *t* = 0 and *S*_m_ is final substrate concentration (g.L^−1^) at time *t*. Kinetics and modeling studies were conducted as in the batch mode.

In the continuous mode, fermentation was also started batch-wise but for 12 h, and then shifted to continuous mode using two simultaneously operated motor pumps to keep feed-rate and product-withdrawal at constant rate to maintain constant volume in the fermentor. To maintain constant reaction volume of 3.5 L, 2.5 L feed was injected into the bioreactor as 2.5 L was withdrawn by means of the motor pumps for 7 continuous runs. An initial optimization of dilution rates was conducted and the best selected on the basis of L-ASNase activity. Analysis was as described for batch and fed-batch modes. Performances of models were evaluated by adjusted goodness-of-fit, *r*^2^, root mean squared error (rmse) and mean absolute error (mse) using the equations detailed in Asitok et al. (2022a).

By considering the costs incurred from purchase of chemicals required to formulate media for fermentation by batch, fed-batch and continuous fermentation modes, and chemicals for post-fermentation analysis; cost of consumable materials as well as labour, the cost incurred from production of l-ASNase by fusant under all three fermentation modes was calculated (Yang and Sha 2022). Energy, equipment and rent costs were omitted since they are known to vary significantly among real-time laboratory arrangements.

### RSM and sensitivity analysis of Fusant-06 L-ASNase purification conditions

By combining Plackett–Burman design (PBD) and response surface methodology (RSM), different molecular weights, concentrations of polyethylene glycol (PEG) and salts, pH and temperature, were screened for significant influences on l-ASNase recovery and purification by aqueous two-phase system (ATPS). Levels of selected significant variables were determined as follows: X_1_ = (Molecular weight—4500)/1500.0; X_2_ = (PEG concentration—15.8)/5.0; X_3_ = (Citrate concentration—20.8)/5.0; X_4_ = (NaCl concentration—12.5)/3.0 and X_5_ = (pH—7.8)/1.0.

In a typical RSM procedure, a sensitivity analysis is conducted to quantify the relationship between the input and output uncertainties in an attempt to determine how sensitive the response model is to fluctuations in the parameters and data on which it is built. This analysis evaluates the robustness of model assumptions and operates how a given model responds to variations in its assumptions (Zhan et al. 2013). In short, it complements design of experiment by revealing non-linear effects of variables.

In the present study, we adopted Sobol method of global sensitivity analysis to evaluate the first-order, second-order and total sensitivity indices of the RSM models using SobolGSA version 2.0 (Imperial College, London). As a continuation, the RSM model was used to generate new parameters using Sobol’ sequence sampling method and the new samples from RSM were run by Sobol method to obtain the sensitivity indices of input parameters. The variance-based method was selected for the Sobol’ sensitivity analysis as it adopts a variance ratio which estimates the importance of each input factor on the response variable. The total variance of the response model is given by the expression below;

4 $$V\left(Y\right)=\sum_{i=1}^{n}{V}_{i}+\sum_{i\le j\le n}^{n}{V}_{ij}+\dots +\sum_{i\le \dots n}^{n}{V}_{1\dots n}$$

where *V* is the total variance of the model output *Y*, *n* is the number of input factors, *V*_*i*_ = first-order effect or partial variance of model output due to *x*_i_, ${{X}_{i}(V}_{i}=V[E\left(Y|{X}_{i}\right)])$ and *V*_ij_ the second-order effect or the partial variance of model output due to interaction between *x*_i_ and *x*_j_, ${{V}_{ij}(V}_{ij}=V\left[E\left(Y|{X}_{i},{X}_{j}\right)\right]-{V}_{i}-{V}_{j})$.

The first-order sensitivity index, *S*_*i*_ was therefore given as a ratio of the first-order effect to the total variance as follows;

5 $${S}_{i}=\frac{{V}_{i}}{V\left(Y\right)}=V[E\left(Y|{X}_{i}\right)])/V(Y)$$

The second-order sensitivity index, *S*_*ij*_ was given as a ratio of the second-order effect to the total variance as follows;

6 $${S}_{ij}=\frac{{V}_{ij}}{V\left(Y\right)}=V\left[E\left(Y|{X}_{i},{X}_{j}\right)\right]-{V}_{i}-{V}_{j})/V(Y)$$

while the total sensitivity index, *S*_*Ti*_ was given by

7 $${S}_{Ti}=\frac{E\left(V\left(Y|{X}_{\sim i}\right)\right)}{V\left(Y\right)}$$

where ~ *i* denotes all input variables except *i* input.

Selected significant factors were employed at their optimum levels for ATPS purification as described by Nascimento et al. (2020). Further purification of the ATPS-extracted protein was performed by molecular exclusion chromatography on Sephadex G-100 of diameter 100 cm by 1.5 cm, using 50 mM phosphate buffer (pH 7.0) and 100 mM KCl as mobile phase at a flow rate of 0.2 mLmin^−1^. Protein standards ranged from 12.4 to 115 kDa (Fisher Scientific Products, India) and comprised cytochrome C (12.4 kDa), soybean trypsin inhibitor (29.0 kDa), ovalalbumin (44.3 kDa), serum albumin (66.2 kDa), phosphorylase *b* (97.2 kDa) and *β*-galactosidase (115 kDa).

### Molecular weight, amino acid profile, substrate specificity, inhibitor, organic solvents, metal ions, temperature and, pH activity and stability characterizations of l-ASNase

Purification level and molecular weight of protein were confirmed by sodium dodecyl sulfate–polyacrylamide gel electrophoresis (SDS-PAGE) with 12% (wv^−1^) gel using the method of Laemmli (1970).

The sequence of amino acids in l-ASNase from Fusant-06 was determined by Agilent amino acid analyzer (Asitok et al. 2022b). Sequences obtained were compared with NCBI sequences for *Aspergillus* l-ASNases and with those of the wild *Aspergillus* strains.

Substrate specificity of l-ASNase was evaluated by incorporating l-asparagine, urea, acrylamide or l-glutamine as enzyme substrates in assay mixture (Imada et al. 1973). l-ASNase activities were expressed relative to activity in the presence of l-asparagine as control.

Effect of enzyme inhibitors reflecting the four classes of protease were evaluated by pre-incubation of fusant l-ASNase with 5 mM and 10 mM of pepstatin A, phenyl-methyl-sulfonyl fluoride (PMSF), di-iso-propyl-fluorophosphate (DPFP), ethylene diamine tetraacetic acid (EDTA), *p*-chloro-mercuric benzoate (*p*CMB), dithiothreitol (DTT), iodoacetamide (IAM), and 5% (vv^−1^) β-mercaptoethanol (β-MEOH) for 15 min. Thereafter, residual activity was determined using assay mixture without inhibitor as control.

Organic solvents, selected based on their octanol–water partition coefficient, log *P* to include toluene, acetonitrile, acetone, cyclohexane, glycerol, ethanol, methanol, *n*-hexane, chloroform, and 2-propanol at 50% (vv^−1^) was studied for their effect on fusant l-ASNase activity at 50 ºC and 200 rpm agitation for 1 h. Thereafter, residual l-ASNase activities were determined using assay mixture without solvent as control.

Influence of metal ions on fusant l-ASNase was evaluated by pre-incubation with 5 mM of cations including K^+^, Na^+^, Ca^2+^, Mg^2+^, Zn^2+^, Mn^2+^, Fe^2+^, Ni^2+^, Cu^2+^, Ba^2+^, Co^2+^, Cr^3+^, Fe^3+^ and Mo^5+^ at 50ºC, pH 7.0 for 15 min before measuring relative activity. Assay mixture without metal served as control.

Effects of temperature (20-70ºC), pH (3–10) and NaCl concentration (5–35%) on enzyme activity were evaluated as described in Ekpenyong et al. (2021a).

### Michaelis–Menten kinetics of Fusant-06 L-ASNase

The Michaelis–Menten constant *K*_m_, maximum velocity, *V*_max_, catalytic rate, *K*_cat_ and catalytic efficiency, *K*_*cat*_*/K*_*m*_ of Fusant-06 l-ASNase were determined by measuring velocity of reaction at varying concentrations of l-asparagine ranging from 0.005–1.28 mM with 5 µg/mL (0.043 µM) l-ASNase and plotting the Lineweaver–Burk relationship between substrate concentration (1/[S]) and reaction velocity (1/V). Catalytic rate was determined by dividing maximum reaction velocity, *V*_max_ by molar concentration of l-ASNase, [E_0_] while the catalytic efficiency was calculated as a ratio of catalytic rate to *K*_m_.

### Potential applications of L-ASNase in health-care and the industry

The MTT-based cytotoxicity assay protocol earlier described (Ekpenyong et al. 2021c) was adopted for in-vitro cytotoxicity of purified l-ASNases against human myeloid leukemia (HL-60), hepatocellular carcinoma (HepG-2) and human breast carcinoma (MCF-7) cell lines. The non-tumor human embryonic cell line (HEK 283 T) was included for selective toxicity evaluation. Test concentrations of all l-ASNases ranged from 0.5 to 1200 µg.mL^−1^. Percent cell viability was determined from triplicate determinations at 570 nm. Data was analyzed by non-linear regression in GraphPad Prism 8 and IC_50_ and selectivity index determined.

For acrylamide reduction potential in food industry, two hundred grams (200 g) of strips of freshly peeled sweet potato (*Ipomoea batatas*), Irish potato (*Solanum tuberosum*) and yam (*Dioscorea esculenta*) were oven-dried for 10 min at 85 ºC and afterwards immersed in 100 U of l-ASNase dissolved in double distilled water. A control preparation was set up without prior exposure to test enzyme and activity determined using Nessler’s reagent (Imada et al. 1973). The preparation was fried in 100 mL of sunflower oil until crunchy strips (chips) were obtained. The strips were ground in a mortar and soaked in 50 mL 90% (vv^−1^) ethanol to extract acrylamide. The ethanol extract was concentrated by evaporation *in vaccuo* and amount of acrylamide (CH_2_ = CHCONH_2_) determined again with Nessler’s reagent.

Antioxidant potential was investigated, in terms of free-radical scavenging potential of the enzyme, using the protocol described by Mihooliya et al. (2020) with ascorbic acid (Sigma-Aldrich, USA) as standard. The change in colour of 2,2-diphenyl-1-picryl-hydrazyl-hydrate (DPPH) from deep violet to pale yellow was monitored spectrophotometrically at a wavelength of 517 nm for 3 h. The scavenging effect was calculated using the expression in Eq. 4 (Syame et al. 2022).

8 $$SE \left(\%\right)=100-\left[\left(\frac{\left({A}_{0}-{A}_{1}\right)}{{A}_{0}}\right)\times 100\right]$$

Additionally, the scavenging activity of Fusant-06 L-ASNase was also tested against the dark blue 2,2ʹ-azino-bis (3-ethylbenzothiazoline-6-sulphonic acid) radical cation (ABTS^.+^) as reported by Liu et al. (2016). Briefly, a solution of decreasing concentrations (µg/mL) of ABTS cation, with an absorbance of 0.705 ± 0.04 at 734 nm, was mixed with ABTS diluent and gently shaken. Subsequently, 10 µL of purified l-ASNase was added to the reaction mixture and left for 6 min in the dark at 28 ± 2ºC (room temperature) and absorbance drop measured at a wavelength of 734 nm using a microplate reader (Thermo Scientific, USA). The scavenging rate (%) was used to evaluate the ABTS scavenging capacity of l-ASNase as described by Liu et al. (2016).

## Results

### Protoplast preparation, fusion and regeneration

Optimum conditions for maximum protoplast isolation were determined by RSM using the Box-Behnken design and results showed that highest number of protoplasts was obtained with mycelial age (X_1_) of 50.5 h, pH (X_2_) of 4.86 and incubation period (X_3_) of 4.2 h (Additional file 1: Figure S1). Under these conditions, a protoplast concentration of 5.98 (± 1.32) × 10^5^ sfu.mL^−1^ was obtained. The regression model was significant at *F* = 82.58, *p* < 0.0001 with an adjusted *r*^2^ of 0.9787 and a LoF test of *F* (3, 7) = 5.15, *p* = 0.0736 > 0.05.

9 $$Y=\,5.53-0.3861{X}_{1}-0.1621{X}_{2}-0.1078{X}_{3}-0.1016{X}_{1}{X}_{2}-0.0768{X}_{1}{X}_{3}-0.0342{X}_{2}{X}_{3}-0.2758{{X}_{1}}^{2}-0.0288{{X}_{2}}^{2}-0.2555{{X}_{3}}^{2}$$

where Y is log concentration of protoplast.

**Fig. 1:**
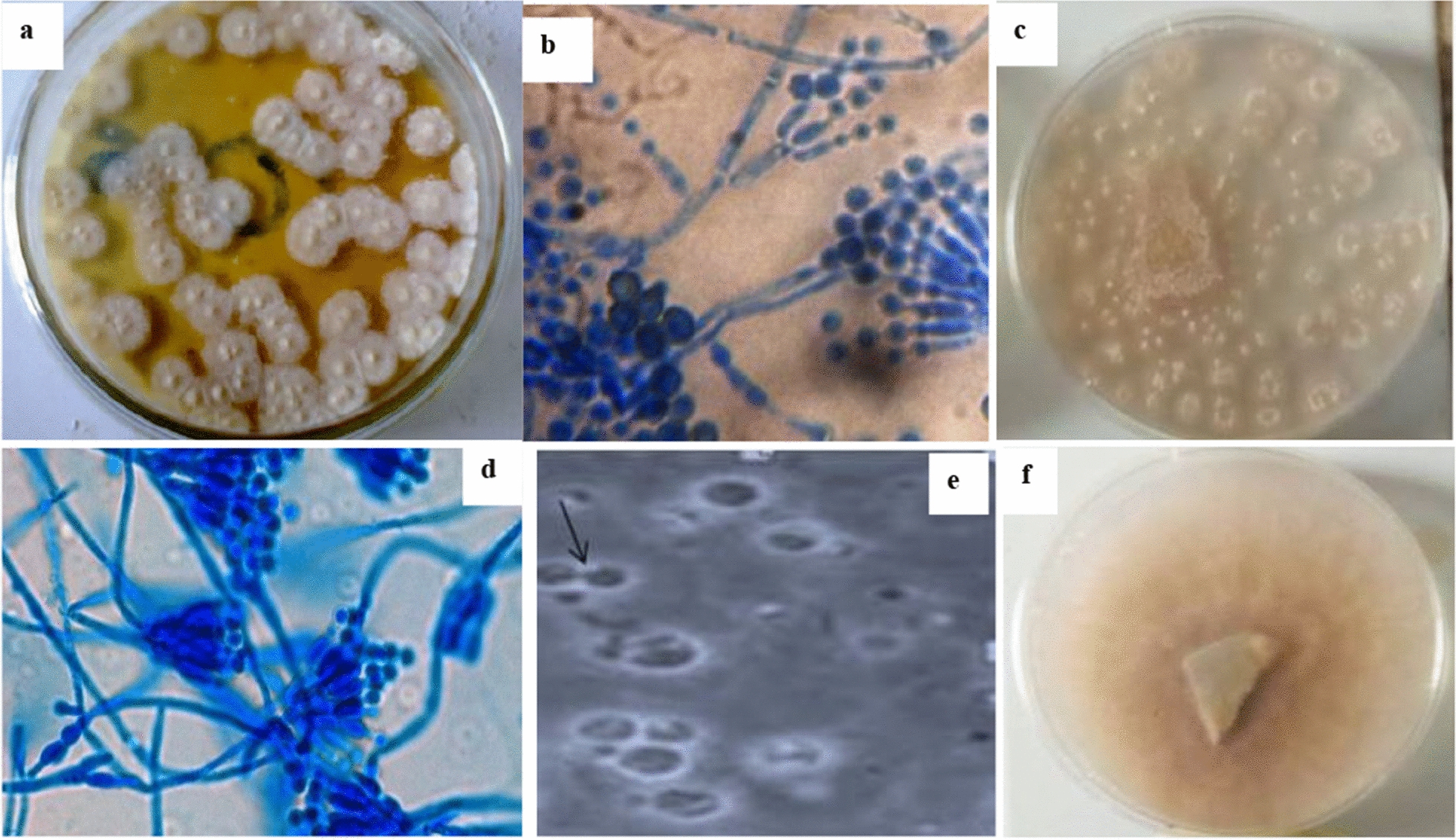
*Aspergillus candidus* and *Aspergillus sydowii* **a**, **c** wild-type strains on PDA plates **b**, **d** in microscopy **e** Fusant-06 in light microcopy 400 X **f** Fusant-06 regenerated on PDA for 72 h

Macroscopic and microscopic observations of protoplast fusion experiment along with wild type strains are illustrated in Fig. 1. Figure 1a and c respectively show 72 h potato dextrose agar (PDA) cultures of wild strains of *Aspergillus candidus* strain UCCM 00,117 and *Aspergillus sydowii* strain UCCM 00,124 while Fig. 1b and d respectively show their cotton-blue in lactophenol microscopy of the strains. Figure 1e and f respectively show fusant formation in microscopy and fusant regeneration of PDA agar.

A total of 13 fusants were isolated and 71.6% of regenerated protoplast fusants showed differing degrees of recombination. Fusant-06 demonstrated highest l-ASNase activity of 24564.74 ± 4093.32 U (Additional file 1: Table S1). Only fusants with xylan-degrading ability, coupled with growth on 25% NaCl PDA at 55ºC, were considered for further studies.

### Comparative techno-economic analysis of L-ASNase production

#### Fermentation type versus fermentation mode

l-ASNase activities of study strains of *Aspergillus* were compared under submerged and solid-state fermentation. Results showed that for all 3 tested strains l-ASNase activity was significantly (*p* < 0.001) higher by submerged fermentation (SmF) than solid state fermentation (SSF). Specifically, Fusant-06 l-ASNase activity by SmF was 1.57-fold higher than that obtained in SSF and so SmF was selected as preferred fermentation type for l-ASNase production by Fusant-06.

Table 1 shows that l-ASNase activity, modeled by the modified Gompertz function (MGM), was 1.43-folds higher in fed-batch fermentation than in batch mode, with a maximum activity of 134364.1 U and 1.2-fold higher than in continuous mode. Biomass concentration (BMC) kinetics, modeled by the logistic function (LGM), revealed that maximum specific growth rate, *µ*_max_ was 3.3-folds higher in the fed-batch mode than in the batch process and 1.32-fold higher than in continuous mode. Substrate consumption was 5.05-fold higher in the fed-batch mode than in the batch and 1.2-folds in the continuous mode. Volumetric rate of substrate consumption, rs_max_ was higher in fed-batch by 3.82-folds than in batch mode but lower than continuous mode by 1.75-fold. However, all calculated yields tilted in favour of the batch process; Yp/s by 3.6-folds higher than fed-batch, 3.53-folds higher than continuous mode; Yx/s by 2.144-folds higher than fed-batch and continuous modes, and Yp/ × 1.68-folds higher than fed-batch and continuous modes.

**Table 1:** Comparative kineto-technical analysis of l-ASNase production by protoplast Fusant-06 under submerged batch, fed-batch and continuous fermentation modes

FM	Biomass formation kinetic parameters by logistic model				Performance statistics of model			
	*X*_0_	*X*_max_	µ_max_	T_d_	Adj. r^2^	RMSE	MAE	*p*-value
Batch	0.034 ± 0.000	876.14 ± 24.35	0.26 ± 0.01	2.67 ± 0.07	0.9987	14.59	11.06	< 0.0001
Fed-batch	0.000 ± 0.00	2061.84 ± 118.06	0.85 ± 0.01	0.815 ± 0.001	0.9995	20.53	10.53	< 0.0001
Con mode	0.01 ± 0.000	1688.72 ± 97.46	0.647 ± 0.01	1.071 ± 0.168	0.9992	20.25	8.34	< 0.0001

FM	l-ASNase formation model parameters by modified Gompertz model			Performance statistics of model			
	*P*_max_	rp_max_	t_L_	Adj. r^2^	RMSE	MAE	*p*-value
Batch	94048.10 ± 3225.32	3653.83 ± 210.84	18.45 ± 2.36	0.9703	8.84	7.11	< 0.0001
Fed-batch	134364.1 ± 7806.24	4454.93 ± 248.35	20.07 ± 2.78	0.9999	77.61	36.10	< 0.0001
Con mode	112251.13 ± 6824.35	3657.56 ± 189.84	19.09 ± 2.87	0.9999	46.73	34.38	< 0.0001

FM	Substrate consumption kinetic parameters by modified Gompertz model								Performance statistics of model			
	*S*_max_	rs_max_	t_L_	Yp/s	Yx/s	Yp/x	q_p_	q_s_	Adj. r^2^	RMSE	MAE	*p*-value
Batch	543.46 ± 32.67	20.33 ± 3.53	23.07 ± 4.25	173.05 ± 28.00	1.612 ± 0.06	107.34 ± 31.32	1.118 ± 0.00	0.007 ± 0.01	0.999	3.21	2.46	< 0.0001
Fed-batch	2742.08 ± 198.64	77.56 ± 9.27	20.62 ± 4.03	48.14 ± 11.28	0.752 ± 0.02	65.17 ± 12.55	0.679 ± 0.06	0.018 ± 0.00	0.980	149.85	115.95	< 0.0001
Con mode	2286.62 ± 202.63	135.94 ± 38.74	20.02 ± 2.75	49.09 ± 9.04	0.756 ± 0.03	64.94 ± 12.27	0.676 ± 0.04	0.014 ± 0.00	0.9974	47.86	31.75	< 0.0001

The kinetic parameter values are means of triplicate determinations ± standard error

*FM* Fermentation mode, *Con mode* Continuous mode, *LGM* Logistic model, *MGM* Modified Gompertz model, P_max_ maximum l-ASNase concentration (mg), *r*_*pmax*_ maximum volumetric rate of l-ASNase formation (mgL^−1^ h^−1^), X_0_ initial biomass concentration (gL^−1^), *X*_*max*_ maximum biomass concentration (gL^−1^), *µ*_*max*_ maximum specific growth rate (h^−1^), *t*_*Lag*_ Lag time (h), *T*_*d*_ Doubling time (h), *S*_*max*_ Maximum predicted substrate consumption (gL^−1^), *r*_*smax*_ maximum volumetric rate of substrate consumption (gL^−1^ h^−1^), *Adj. r*^*2*^ adjusted coefficient of determination, *RMSE* root mean squared error, *MAE* mean absolute error, *Yp/s* l-ASNase yield relative to amount of substrate consumed, *Yx/s* Biomass yield relative to amount of substrate consumed, *Yp/x* Specific l-ASNase activity, *qp* specific rate of l-ASNase activity, *qs* Specific rate of substrate consumption, *Qp* Volumetric productivity of l-ASNase, U(L^−1^.h^−1^), *Q*_*x*_ Volumetric biomass productivity, g DCW(L^−1^.h.^−1^)

### Comparative cost analysis of l-ASNase production

Results of cost analysis for all fermentation modes are presented in Table 2 and show that cost of operating fed-batch was low compared to batch and continuous modes of production. Since Pearson’s bivariate correlation results consistently indicated strong correlation (*r* = 0.960) between l-ASNase activity and biomass concentration (gram dry cell weight, gDCW), cost of production was evaluated using quantification of dry cell weight (DCW). Specifically, $1.38 was expended per gDCW of Fusant-06 in the fed-batch mode, $1.85 and $2.65 in batch and continuous fermentation modes, respectively.

**Table 2:** Cost analysis of fermentation modes towards l-ASNase production by *Aspergillus* protoplast Fusant-06 and wild strains in a 3.5 L working volume bioreactor

Cost parameters	l-asparaginase producing strains under differing fermentation modes								
	Protoplast fusant			*Aspergillus candidus*			*Aspergillus sydowii*		
	Batch	Fed-batch	Continuous	Batch	Fed-batch	Continuous	Batch	Fed-batch	Continuous
Chemicals ($)	14.8	24.5	16.8	14.8	24.5	16.8	14.8	24.5	16.8
Consumables ($)	750	1150	1025	750	1150	1025	750	1150	1025
Labour ($50 h^−1^)	4900	10000	15000	5000	10000	15000	5000	10000	15000
Sum ($)	5664.8	11174.5	16041.8	5764.8	11174.5	16041.8	5764.8	11174.5	16041.8
DCW per vessel (g)	3066.49	8122.59	6050.065	2475.0	5294.34	4228.25	2599.24	4677.42	3938.45
Production cost per DCW ($g^−1^)	1.85	1.38	2.65	2.33	2.11	3.79	2.22	2.39	4.07

*DCW* Dry cell weight; chemicals comprised media components employed to formulate production media, feeding nutrients and dilution nutrients; consumables comprised; labour cost comprised the cost of medium preparation, sterilizing, operating, cleaning, washing of bioreactors, and analysis of products (three employees for continuous mode, 2 for fed-batch and 1 for batch @50 $h^−1^ multiplied by 100 h; DCW per 5 L vessel with 3.5 L working volume; Total fermentation time = 100 h, from preparation through running to clean up

### Response surface optimization of l-ASNase purification by aqueous two-phase system (ATPS) and sensitivity analysis

Five significant factors including molecular weight of polyethylene glycol (PEG—X_1_), concentration of PEG (X_2_), concentration of citrate (X_3_), concentration of NaCl (X_4_) and pH (X_5_), selected by PBD were modeled by RSM to obtain maximum yield (Y_1_) and purification (Y_2_) of L-ASNase as follows;.

10 $${Y}_{1}=\,66.29+9.3{X}_{1}+4.61{X}_{2}-5.34{X}_{3}+1.55{X}_{4}-3.79{X}_{5}+2.18{X}_{1}{X}_{2}-3.02{X}_{1}{X}_{3}-4.36{X}_{1}{X}_{4}+3.06{X}_{1}{X}_{5}-1.8{X}_{2}{X}_{3}+0.72{X}_{2}{X}_{4}+1.76{X}_{2}{X}_{5}-0.01{X}_{3}{X}_{4}+8.05{X}_{3}{X}_{5}-2.98{X}_{4}{X}_{5}-1.52{{X}_{1}}^{2}-1.81{{X}_{2}}^{2}-3.18{{X}_{3}}^{2}-4.93{{X}_{4}}^{2}-0.04{{X}_{5}}^{2}$$

11 $${Y}_{2}=\,29.58-1.97{X}_{1}+3.81{X}_{2}+4.37{X}_{3}-3.2{X}_{4}-1.51{X}_{5}+4.92{X}_{1}{X}_{2}-11.74{X}_{1}{X}_{3}+2.23{X}_{1}{X}_{4}-6.24{X}_{1}{X}_{5}-5.05{X}_{2}{X}_{3}-1.28{X}_{2}{X}_{4}+0.53{X}_{2}{X}_{5}-1.42{X}_{3}{X}_{4}+7.73{X}_{3}{X}_{5}-0.73{X}_{4}{X}_{5}+3.08{{X}_{1}}^{2}-1.76{{X}_{2}}^{2}+2.88{{X}_{3}}^{2}+1.72{{X}_{4}}^{2}+0.554{{X}_{5}}^{2}$$

**Fig. 2:**
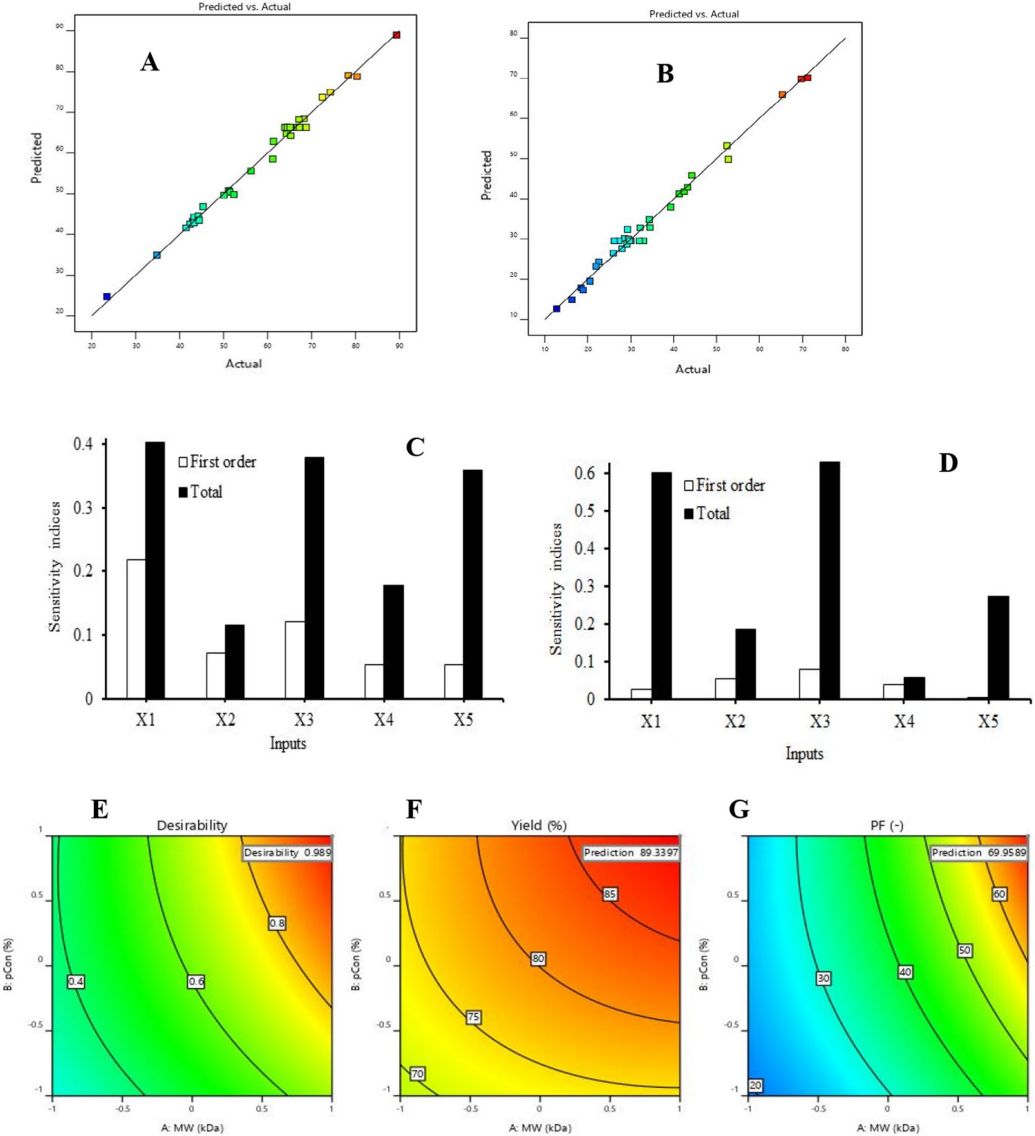
Multi-objective response surface optimization of conditions for aqueous two-phase system (ATPS) extraction and purification of l-ASNase from fermentation broth: **A** & **B** are model performance plots of actual versus predicted yields and purification factors, respectively; **C** & **D** are first and total order global sensitivity indices for yield and purification factor, respectively; **E** is the desirability contour (2D) plot for the multi-objective optimization while **F** & **G** are the optimized contour plots for yield and purification factor, respectively

Analysis of variance (ANOVA) of the regression models revealed that both models were significant at *p* < 0.05; adjusted *r*^2^ of 0.9798, lack-of-fit (LoF) = 0.3332 for Y_1_ (Fig. 2a) and adjusted *r*^2^ of 0.9669, LoF = 0.4979 for Y_2_ (Fig. 2b) suggesting that they were adequate for prediction of optimum conditions for the purification process.

Global sensitivity analysis provides a better understanding of the sensitivity of the outputs (yield and purification factor) to the inputs (molecular weight, polymer concentration, citrate concentration, NaCl concentration and pH). The first and total order sensitivity indices determined using the Sobol variance decomposition method for yield and purification factor are presented in Fig. 2c and d respectively. The model representing yield (Fig. 2c) shows that the relative importance of the input parameters could be ranked as follows: molecular weight > citrate concentration > polymer concentration > pH > NaCl concentration. Molecular weight, with a total sensitivity index of 0.40, was the most sensitive parameter while NaCl concentration, with a total sensitivity index of 0.18 was the least sensitive parameter. On the contrary, for the model representing purification factor (Fig. 2d), the order was as follows: citrate concentration > polymer concentration > NaCl concentration > molecular weight > pH. Citrate concentration was the most sensitive parameter with a total sensitivity index of 0.63 while pH was not a sensitive parameter having a total sensitivity index of 0.06 which is less than the benchmark of 0.1.

The RSM models were optimized using the desirability function of multi-objective optimization and conditions for purification were set at (1, −1, 1, 1, −1) = (X_1_, X_2_, X_3_, X_4_, X_5_) corresponding respectively in actual values to PEG-6000 kDa, 20.8% (w.v^−1^) PEG, 15.8% (w/v) sodium citrate, 12.4% (w/v) NaCl at a pH of 6.8 at a composite desirability, *D* of 0.989. Optimum interaction conditions between citrate concentration and pH produced maximum Y_1_ and interaction between molecular weight of PEG and citrate concentration produced maximum Y_2_. At a composite desirability, *D*, of 0.989 (Fig. 2e), these conditions resulted in l-ASNase yield of 89.34% (Fig. 2f) and a purification factor of 69.96 (Fig. 2g, Table 3).

**Table 3:** Aqueous two-phase system (ATPS) purification of Fusant-06 l-ASNase

Purification step	PEG Mol. wt	Total protein (mg)	Total activity (U)	Specific activity (Umg^−1^)	Yield (%)	Fold purification
Crude extract	–	1926.400	132007.00	68.525	100.00	1.00
ATPS	6000	24.601	117935.05	4793.91	89.34	69.96
Sephadex G-100	–	8.036	80511.07	10019.04	60.99	146.21

PEG-6000/Na^+^ Citrate (20.8% PEG 6000, 15.8% sodium citrate, 12.4% NaCl and pH 6.8

*PEG* Polyethylene glycol, *Mol. wt* Molecular weight, *ATPS* Aqueous two-phase system

### Molecular weight, amino acid profile, substrate specificity, inhibitor, organic solvents, metal ions, temperature and, pH activity and stability characterizations of l-ASNase

**Fig. 3:**
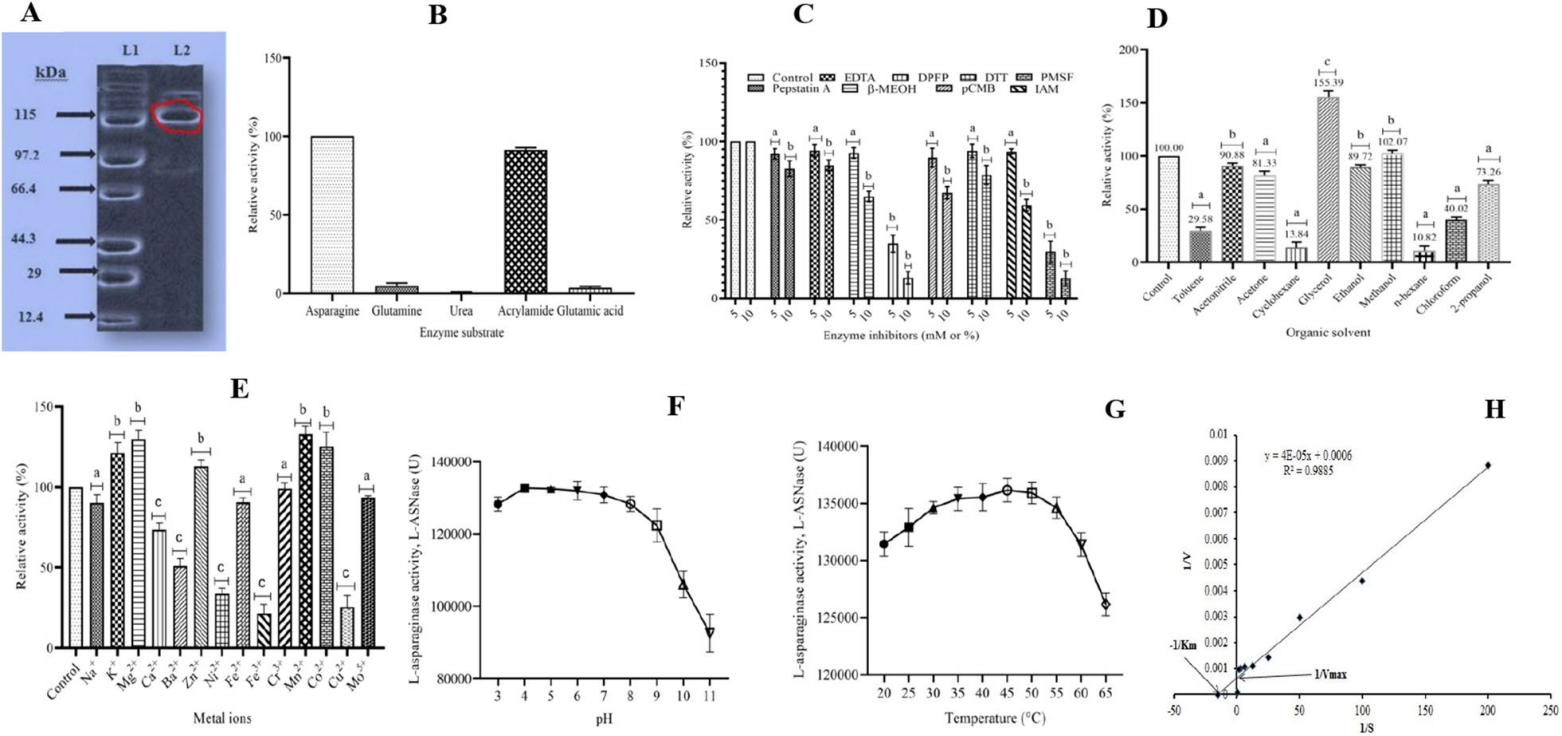
Characterization of Fusant-06 L-ASNase **A** SDS-PAGE molecular weight analysis; Lane 1 (**L1**) shows protein standards and Lane 2 (**L2**) the purified Fusant-06 L-ASNase **B** Substrate specificity **C** Inhibitor stability—bars with letter ‘a’ are significantly different from control; bars with letter ‘b’ are not significantly different from control **D** Organic solvent stability **E** metal ions **F** pH **G** temperature enzyme activity **H** Michealis –Menton kinetics of Fusant-06 l-ASNase. Values are means of triplicate determinations and error bars are standard deviations tested at 5% significance level. Error bars with letter ‘a’ are not significantly different from control; bars with letter ‘b’ significantly enhance l-ASNase activity; bars with letter ‘c’ significantly inhibit l-ASNase

The 69.96-fold ATPS-purified L-ASNase subjected to molecular exclusion chromatography yielded 146.21-fold purification for an 8.04 mg protein with 60.99% yield. The molecular weight was determined with molecular exclusion chromatography where an elution ratio of 0.587 for fusant l-ASNase was fitted into the equation; $y=-1.7519x+3.0946$ (adjusted *r*^2^ = 0.9203), obtained by regressing elution ratio on log molecular weight of standard proteins (Additional file 1: Figure S3). This gave a molecular weight of 116.4 kDa which was confirmed by SDS-PAGE (Fig. 3a). The amino acid sequence of Fusant-06 l-ASNase showed a length of 513 residues with those of recombining wild type strains given as 378 (72.64 kDa) for Asp-C and 225 (44.36 kDa) for Asp-S (Additional file 1: Figure S4).

Figure 3b illustrates substrate specificity of Fusant-06 L-ASNase where urea (0.56%), glutamine (8.56%) and glutamic acid (4.21%) substrates showed very low affinity for the enzyme compared to l-asparagine. Acrylamide, on the other hand, demonstrated significant (87.25%, *p* < 0.05) affinity for the enzyme relative to l-asparagine.

Figure 3c reports the results of enzyme inhibition by chemical compounds and shows that 10 mM or 10% (vv^−1^) concentration of all chemicals significantly inhibited Fusant-06 l-ASNase activity more than 5 mM concentrations. Enzyme activities after pre-exposure to 5 mM of pepstatin A, EDTA, pCMB, β-MEOH, DTT and IAM were not significantly (*p* > 0.05) inhibited as the enzyme retained ≥ 92% of its activity. However, l-ASNase activity was significantly inhibited by serine-protease inhibitors namely PMSF and DPFP even at their low concentrations.

Results of the effect of organic solvents on Fusant-06 L-ASNase activity showed that acetonitrile, toluene, ethanol, methanol and 2-propanol had no significant (*p* < 0.05) effect on l-ASNase activity (Fig. 3d). However, while acetone, cyclohexane, *n*-hexane and chloroform significantly (*p* < 0.05) inhibited enzyme activity, glycerol significantly (*p* < 0.05) enhanced it to 134.74% relative to control.

The activity of Fusant-06 l-ASNase was enhanced tremendously in the presence of Mn^2+^ (184.60%). Figure 3e shows that K^+^, Mg^2+^, Co^2+^ and Zn^2+^ also significantly enhanced activity to 151.19, 129.64, 125.27 and 112.99% respectively. Contrariwise, Ca^2+^, Ba^2+^, Ni^2+^, Fe^3+^ and Cu^2+^ significantly (*p* < 0.05) inhibited enzyme activity with Fe^3+^ showing the most inhibition of 31.62%. Na^+^, Fe^2+^, Cr^3+^ and Mo^5+^ did not significantly (*p* > 0.005) influence enzyme activity as  ≥ 90% of activity was retained after respective pre-exposures.

pH optimum of Fusant-06 l-ASNase activity ranged from pH 3 to 9 as Tukey’s multiple comparison tests showed no significant difference (*p* > 0.05) among those pH levels (Fig. 3f).

Influence of temperature is illustrated in Fig. 3g and Tukey’s multiple comparison tests showed significant stable optimum over a wide range of temperatures from 20 to 60 ºC.

Fusant-06 l-ASNase tremendously tolerated exposure to high NaCl concentrations up to 30% (Additional file 1: Figure S6).

### Michaelis–Menten kinetics of Fusant-06 L-ASNase

The kinetic parameters of fusant-06 L-ASNase were determined from Lineweaver–Burk plot of 1/V against 1/[S] in Fig. 3h. The *K*_m_ was determined as 6.67 × 10^–5^ M, V_max_ as 1666.67 µmol.min^−1^, K_cat_ calculated with 5 µg.mL^−1^ (0.043 µM) of L-ASNase [E_0_] as 3.88 × 10^4^ min^−1^ and K_cat_/K_m_ as 5.81 × 10^8^ M^−1^.min^−1^.

### Potential applications of fusant-06 l-ASNase

**Fig. 4:**
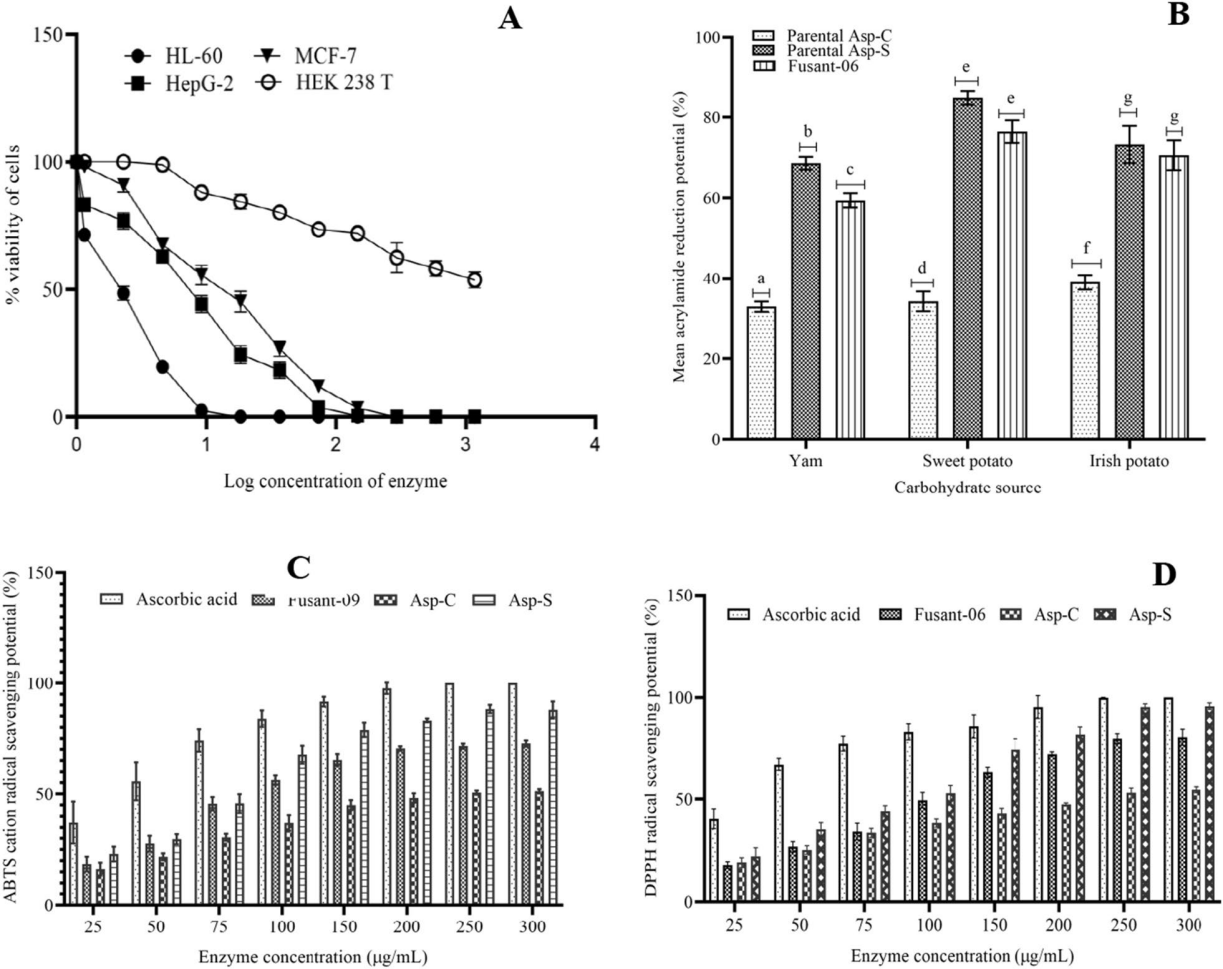
Cytotoxic potential (**A**), acrylamide reduction potential (**B**), concentration-dependent ABTS (**C**) and DPPH (**D**) free-radical scavenging potential of l-ASNases; values are means of triplicate determinations and error bars are standard deviations. Bars with similar alphabets are not statistically different from each other while those with dissimilar letters are statistically different from each other at significance level of 5%

Studies to investigate the anti-leukemic potential of study l-ASNase revealed that cytotoxicity was dose-dependent and increased significantly as enzyme concentration increased (Fig. 4a). The human leukemia cell line (HL-60) was most susceptible to the enzyme as only 1.055 µgmL^−1^ of the enzyme was required to reduce cell viability by 50% (Table 3). Inhibition concentration 50 (IC_50_) of fusant-06 l-ASNase against HL-60 cell line improved by 3.01-fold from Asp-C l-ASNase. When compared with other fungal l-asparaginases in our laboratory, fusant-06 enzyme outperformed others in terms of IC_50_ values. The selectivity index of fusant-06 l-ASNase against HL-60 was more than 6.0-folds higher than observed for other cancer cell lines as shown in Table 4.

**Table 4:** Indicators of therapeutic potentials of protoplast Fusant-06 l-ASNase

Strain	Cell line	IC_50_ (µg/mL)	SI	Adjusted *r*^2^	RMSE
Fusant		1.055	77.13	0.9775	4.782
Ile^−^Thr^−^Asp-C-180-K	HL-60	3.178	27.66	0.9879	1.739
Val^−^Asp-S-180-E		19.738	4.98	0.9877	0.763
Fusant		6.359	12.80	0.9865	4.035
Ile^−^Thr^−^Asp-C-180-K	HepG-2	9.392	9.36	0.9648	3.119
Val^−^Asp-S-180-E		22.683	4.34	0.9463	0.872
Fusant		9.446	8.61	0.9910	3.528
Ile^−^Thr^−^Asp-C-180-K	MCF-7	11.831	7.43	0.9934	1.928
Val^−^Asp-S-180-E		25.458	3.86	0.9884	2.172
Fusant		81.370	–	0.9670	2.905
Ile^−^Thr^−^Asp-C-180-K	HEK-238 T	87.890	–	0.9898	1.283
Val^−^Asp-S-180-E		98.361	–	0.9789	1.663

*HL-60* Human myeloid lymphoma, *HepG-2* Hepatocellular carcinoma, *MCF-7* Human breast carcinoma

Figure 4b shows that acrylamide reduction potential (ARP) of Fusant-06 l-ASNase was a function of carbohydrate source with highest ARP observed for potato. Fusant-06 l-ASNase ARP of 76.46% was intermediate between those from wild strains against sweet potato chips but significantly lower than recombining wild strains in yam chips. Tukey’s multiple comparison’s test at 95% confidence limit showed that there was no statistically significant difference between Irish and sweet potato acrylamide reduction potentials of Fusant-06 and Asp-S l-ASNases. However, significant differences existed between Asp-C and Fusant-06 as well as Asp-C and Asp-S L-ASNases.

The antioxidant potential of Fusant-06 l-ASNase against both radicals of ABTS cation and DPPH increased with increasing concentration of the enzyme up until 250 µgmL^−1^ where a constant mean antioxidant activity of 71.51% was observed against ATBS radical compared to 100% free-radical scavenging potential (FRSP) of ascorbic acid (control) at that concentration (Fig. 4c). DPPH free-radical scavenging potential of Fusant-06 l-ASNase at that concentration was ~ 80% compared to ~ 95% by Asp-S L-ASNase (Fig. 4d). Against ATBS and DPPH radicals, Asp-C l-ASNase scavenging potentials at 250 µg/mL were ~ 51 and 53%, respectively.

## Discussion

Mycelial age is significant for protoplast isolation and regeneration because while young mycelia require short time mild treatments, old mycelia may require prolonged incubations for enzymatic digestion of cell walls leading frequently to loss of nuclei which may affect protoplast regeneration. Ushijima and Nakadai (1987) reported a regeneration ratio greater than 35% in their study on Koji molds by intraspecific protoplast fusion of *Aspergillus sojae* strains which was not significantly different from the 37.3% regeneration ratio in this study.

The preference for SmF by Fusant-06 to produce l-ASNase was not surprising because wild type strains preferentially produced 6-times more l-ASNase in SmF than SSF (Ekpenyong et al. 2021a; unpublished data). Industrial production of l-ASNase has been mostly performed by SmF mainly from the point of view of higher yield (da Cunha et al. 2022). In the 96-h fermentation in this study, SmF significantly outperformed (*p* < 0.0001) SSF in all tested strains.

In comparing SmF fermentation modes, fed-batch fermentation was selected from technical and economic viewpoints. Technically, volumetric l-ASNase productivity, the main driver for industrial productions, was highest by fed-batch mode. Most industrial fermentations with molds are conducted by fed-batch mode especially when product yield strongly correlates with biomass (Yang and Sha 2022). The grey areas against batch and continuous fermentation modes in this study were maximum specific growth rate, *µ*_max_ and labour cost respectively. With a *µ*_max_ of only 0.26 h^−1^ in batch culture, fusant population doubled, T_d_ every 2.67 h but every 1 h in continuous culture with a *µ*_max_ was 0.647 h^−1^. Economically, production cost per bioreactor volume still favoured fed-batch mode as the cheapest to run, with an average production cost per gram Fusant-06 DCW of $1.38. While only one worker could operate the bioreactor in batch mode, it took three workers to efficiently operate continuous mode to meet up with overall fermentation time, otherwise unproductive idle time would reduce overall volumetric productivity, Qp.

Optimized conditions for maximum extraction and purification of Fusant-06 l-ASNase by PEG/Citrate ATPS gave a purification factor of 69.96 and yield of 89.34%. The primary step required further purification by chromatography to achieve higher purification of 146.23-fold. Santos et al. (2018) reported extraction of 87.94% and 20.09-fold purified periplasmic l-ASNase from *Escherichia coli* with ATPS using PEG/Citrate system under optimized conditions. Primary (one-step) purification of other microbial products by PEG/Citrate aqueous two-phase system has also been reported by Flores-Gatica et al. (2021) who recovered hyaluronic acid with 79.4% recovery and 74.5-fold purification factor. However, pre-purification of crude extract by ammonium sulfate precipitation before ATPS application significantly improved purification by 173.8-fold.

Sensitivity analysis is important in that it provides insight into the identification of the contribution of model parameters to the uncertainty in the model output (Amenaghawon et al. 2022). In this regard, the most sensitive parameters are identified. The first order indices give an indication of the contribution of the first order (single effect terms) parameters to the output variance while the total order indices measure the combined contribution of the single effect terms and their interaction to the variance of the output (Xu et al. 2018). In taking a sensitive parameter as one with a total order sensitivity index greater than 0.1, it was found that all the input parameters were sensitive for the model representing yield (Zhang et al. 2016). This is supported by the observation recorded from the ANOVA results which indicated that all the inputs were significant with *p* values less than 0.05. However, the limitation of ANOVA is that it does not indicate the actual contribution of each individual term to the variance of the model output. Our study revealed that attention should be given to molecular weight of the polymer when designing processes for extracting l-ASNase by ATPS to optimize yield. The purification processes for l-ASNase required careful manipulation of citrate concentration. The difference between the first order and total order indices accounted for the greatest percentage of the total order indices indicating strong interaction between the input parameters.

Molecular weight of l-ASNase varies between 33 and160 kDa (Cardoso et al. 2020) which accommodates the 116.4 kDa of Fusant-06 l-ASNase in this study. An l-ASNase with molecular weight of 115 kDa from *Aspergillus oryzae* CCT 3940 has been reported by Dias et al. (2016). The amino acid sequence length of 513 residues was almost the sum of the component parental strains suggesting significant genome recombination (Strom and Bushley 2016). However, the significant increase in molecular weight could be attributed to the increased presence of high molecular weight amino acid residues like tryptophan, tyrosine, and aspartic acid. In terms of polarity, the distribution of positively and negatively charged amino acids was such that gave the protein good coverage for acid and alkali tolerance. Comparatively, the ARTP mutagenesis introduced high molecular weight amino acid residues in Asp-C resulting in higher weight of the protein. On the contrary, the same mutation caused sequence shrinkage in Asp-S resulting in lower molecular weight protein than the parent strain but with higher l-ASNase activity.

Cross-reactivity of l-ASNase with glutamine and urea is the cause of most untoward reactions during therapy. The low glutaminase and urease activities of Fusant-06 l-ASNase confer safety on the product thus paving way for clinical applications (Prakash et al. 2020). Glutamine is the dominant nitrogen source in serum and its undesirable hydrolysis by l-ASNase during cancer chemotherapy could lead to adverse events. However, a few studies have demonstrated that low glutaminase activity, such as obtained in this study, could prolong anti-leukemic activity of l-ASNase (Chan et al. 2019). Blood urea required for buffering could also be hydrolyzed by some l-ASNases similarly leading to clinical complications. Undesirable hydrolysis of blood urea by urease results in the release of carbon dioxide which upon dissolution in the aqueous environment forms the bicarbonate ion required for buffering (Doriya and Kumar 2016). However, the strong affinity of Fusant-06 l-ASNase for acrylamide is exploited in the food industry for treatment of high-content carbohydrate foods prior to baking or frying.

Only serine protease inhibitors including phenyl-methyl-sulfonyl fluoride (PMSF) and di-iso-propyl-fluorophosphate (DPFP) could inhibit l-ASNase activity suggesting that Fusant-06 l-ASNase is a serine protease. This agrees with El-Gendy et al. (2021) who reported similar inhibition pattern for *Fusarium equiseti* AHMF4 l-ASNase.

Stability of Fusant-06 l-ASNase to organic solvents strongly and significantly correlated with the octanol–water partition coefficients, log *P* (Sangster 1989) of the solvents (Additional file 1: Figure S5), suggesting that enzyme stability reduced with increase in solvent hydrophobicity (Asitok et al. 2022b).

Fusant-06 l-ASNase activity was enhanced by Mn^2+^, K^+^ and Mg^2+^ much like parent strains (Ekpenyong et al. 2021a; unpublished data). The significant inhibition of l-ASNase activity by Cu^2+^ and Ni^2+^ at 5 mM is corroborated by the work of El-Naggar et al. (2018) on *Streptomyces brollosae* strain NEAE-115 l-ASNase and goes to suggest increased affinity of the enzyme for its substrate (l-asparagine) Mn^2+^ and Mg^2+^ ions.

The significant difference in temperature and pH optima of Fusant-06 l-ASNase and its parents is that they are broader in the fusant. *Aspergillus candidus* strain UCCM 00117 and *Aspergillus sydowii* strain UCCM 00124 had their temperature optima at 50 ºC (Ekpenyong et al. 2021a; unpublished manuscript) and were inhibited at 30 and 70 ºC. However, Fusant-06 l-ASNase demonstrated 97% of total activity from 20 to 60 ºC and 94.5% activity from pH 3 to 9. Vala et al. (2018) reported a broad pH range of 4.0–10.0 for *Aspergillus niger* strain AKV-MKBU l-ASNase. The excellent stability of Fusant-06 l-ASNase at 60 ºC is significant and useful for application of the enzyme in the food and pharmaceutical industries (Dias et al. 2019). Very few l-asparaginases, if at all, can tolerate NaCl concentration as high as 30% as demonstrated by Fusant-06 l-ASNase. This suggests strong stability of molecular structure of the enzyme which may confer significant applicability in treatment of salted food products.

A V_max_ of 1666.67 µmol.min^−1^ by Fusant-06 l-ASNase suggests that 1666.67 µmol of NH_3_ is generated in 1 min at saturation point. Saeed et al. (2021) reported a V_max_ of 535.5 µmoles.min^−1^ for *Dickeya chrysanthemi* L-ASNase but Li et al. (2018) reported a higher V_max_ of 2929 µmol.min^−1^ for *Pyrococcus yayanosii* CH1. The corresponding low K_m_ of 1.667 × 10^–3^ M in this study indicates high substrate affinity which allows therapeutic depletion of l-asparagine during therapy. The K_m_ of Fusant-06 l-ASNase is lower than 5.6 × 10^–3^ M of *Pyrococcus yayanosii* (Li et al. 2018) and the 1.01 × 10^–2^ M of *Streptomyces fradiae* NEAE-82 l-ASNase (El-Naggar et al. 2016). However, the l-ASNases of *Bacillus licheniformis* and *Vibrio succinogenes* have comparatively lower K_m_ of 1.4 × 10^–5^ M (Kafkewitz and Goodman 1974) and 1.7 × 10^–5^ M (Mahajan et al. 2012) respectively. The K_cat_ or turnover value of Fusant-06 l-ASNase suggests that ~ 6.46 × 10^2^ molecules of l-asparagine were converted to product in one second per molecule of enzyme. This makes the l-ASNase commendably efficient as given by the specificity constant K_cat_/K_m_ of 387522.50 M^−1^.s^−1^.

The cytotoxic potential of Fusant-06 l-ASNase towards human myeloid lymphoma (HL-60), hepatocellular carcinoma (HepG-2) and human breast carcinoma (MCF-7) cell lines were all in dose-dependent manner which warranted the determination of the inhibition concentration 50 (IC_50_) against the cell lines. The fusant l-ASNase demonstrated most toxicity against HL-60 with an IC_50_ of 1.055 µg.mL^−1^ and a selectivity index of 77.13 indicating preference for treatment of lymphoblastic leukemia. Hassan et al. (2018) reported preferential cytotoxicity of *Aspergillus terreus* l-ASNase (IC_50_ = 3.27 µg.mL^−1^) against HepG-2 cells.

Acrylamide is described as a potential carcinogen with asparagine as precursor. Prior treatment of sweet potato with Fusant-06 l-ASNase before frying grossly reduced acrylamide content (76.46%) through hydrolysis of l-asparagine into aspartic acid that could not engage in acrylamide formation (Jia et al. 2021). This result is corroborated by Mahajan et al. (2012) who reported acrylamide reduction by 80% in fried potato strips using *Bacillus licheniformis* l-ASNase.

Free-radicals are the primary predisposing factors to several forms of cancer where they occur frequently as reactive oxygen species. Their primary mechanism of action is induction of oxidative stress in cells by short-circuiting the flow of oxygen. Fusant-06 l-ASNase showed a dose-dependent free-radical (DPPH and ATBS^.+^) scavenging activity with peak activity of 84% observed at 300 µg.mL^−1^ in 90 min. El-Gendy et al. (2021) reported a similar dose-dependent free-radical scavenging potential for *Fusarium equiseti* AHMF4 l-ASNase.

From the foregoing discussion, it can be concluded that protoplast fusion of *Aspergillus* strains enhanced the anti-leukemic potential of *Aspergillus candidus* strain UCCM 00117 and the acrylamide reduction and antioxidant potentials of *Aspergillus sydowii* strain UCCM 00124 l-ASNases. The 116.4 kDa protein, recovered primarily using RSM and global sensitivity model optimized conditions of aqueous two-phase system, was identified as a 513 residue serine protease with wide pH, temperature optima and salt tolerance. Additionally, its phenomenal catalytic efficiency, commendable acrylamide reduction and antioxidant potentials make it suitable for economic and reliable clinical and food industry applications.

## Supplementary Information

**Additional file 1: Table S1. **Growth factor combinations for biochemical mutant characterization. **Figure S1.** Box-Behnken designed (BBD) response surface optimization (RSM) of conditions for maximum isolation of protoplasts. **Table S2.** Protoplast fusant L-asparaginase screening. **Figure S2.** Comparative evaluation of L-asparaginase production by fusant-09, Asp-C (*Aspergillus candidus*) and Asp-S (*Aspergillus sydowii*) in (A) submerged (SmF) and solid-state fermentation (SSF); (B) batch kinetics; (C) fed-batch kinetics (D) continuous fermentation kinetics. **Figure S3.** Determination of Fusant-06 L-ASNase molecular weight by plotting log molecular weight of standard proteins against elution ratio and solving the regression equation using the elution ratio of the test L-ASNase (0.587). **Figure S4.** Amino acid profiles of the L-asparaginases from study *Aspergillus* strains. **Figure S5.** Relationship between octanol-water partition coefficients of organic solvents and relative ASNase activity. **Figure S6.** Effect of NaCl on L-asparaginase activity.

## Data Availability

The datasets generated and/or analyzed during the current study are available from the corresponding author on reasonable request.
