# Supplementary material for: Production, characterization and techno-economic evaluation of Aspergillus fusant l-asparaginase
Source: AMB Express. 2023 Jan 6;13:2. doi: 10.1186/s13568-022-01505-8 (PMC9823191; doi:10.1186/s13568-022-01505-8)
Supplement: Supplementary file 1 — Additional file 1: Table S1. Growth factor combinations for biochemical mutant characterization. Figure S1. Box-Behnken designed (BBD) response surface optimization (RSM) of conditions for maximum isolation of protoplasts. Table S2. Protoplast fusant L-asparaginase screening. Figure S2. Comparative evaluation of L-asparaginase production by fusant-09, Asp-C (Aspergillus candidus) and Asp-S (Aspergillus sydowii) in (A) submerged (SmF) and solid-state fermentation (SSF); (B) batch kinetics; (C) fed-batch kinetics (D) continuous fermentation kinetics. Figure S3. Determination of Fusant-06 L-ASNase molecular weight by plotting log molecular weight of standard proteins against elution ratio and solving the regression equation using the elution ratio of the test L-ASNase (0.587). Figure S4. Amino acid profiles of the L-asparaginases from study Aspergillus strains. Figure S5. Relationship between octanol-water partition coefficients of organic solvents and relative ASNase activity. Figure S6. Effect of NaCl on L-asparaginase activity. [file 13568_2022_1505_MOESM1_ESM.docx]

**SUPPLEMENTARY MATERIAL**

**Production, characterization and techno-economic evaluation of *Aspergillus* fusant L-asparaginase**

Atim Asitok^1,2^, Maurice Ekpenyong^1,2*^, Andrew Amenaghawon^3^, Ernest Akwagiobe^4^, Marcus Asuquo^5^, Anitha Rao^4^, David Ubi^4^, Juliet Iheanacho^1^, Joyce Etiosa^1^, Agnes Antai^6^, Joseph Essien^7^ & Sylvester Antai^1,2^

1. Environmental Microbiology and Biotechnology Unit, Department of Microbiology, University of Calabar, Nigeria
2. University of Calabar Collection of Microorganisms (UCCM), Department of Microbiology, University of Calabar, Nigeria
3. Department of Chemical Engineering, University of Benin, Benin City, Nigeria
4. Industrial Microbiology and Biotechnology Unit, Department of Microbiology, Faculty of Biological Sciences, University of Calabar, Nigeria
5. Department of Hematology, University of Calabar Teaching Hospital, Calabar, Nigeria
6. Department of Economics, Faculty of Social Sciences, University of Calabar, Nigeria
7. Department of Microbiology, Faculty of Science, University of Uyo, Nigeria

*Corresponding author: [maurygg2002@yahoo.com](mailto:maurygg2002@yahoo.com); [mauriceekpenyong@unical.edu.ng](mailto:mauriceekpenyong@unical.edu.ng) ; +234-70-38218972

**Table S1 Growth factor combinations for biochemical mutant characterization**

| **Plates** | **Plates** | | | | | |
| --- | --- | --- | --- | --- | --- | --- |
|  | **1** | **2** | **3** | **4** | **5** | **6** |
| **7** | Adenine | Biotin | Phenylalanine | Alanine | Arginine | Leucine |
| **8** | Hypoxanthine | Folic acid | Serine | Cysteine | Ornithine | Glycine |
| **9** | Cytosine | Pantothenic acid | Tryptophan | Threonine | Aspartic acid | Isoleucine |
| **10** | Guanine | Pyridoxine | Tyrosine | Thiosulfate | Proline | Histidine |
| **11** | Thymine | Thiamin | *p*-amino benzoic acid | Methionine | Glutamic acid | Lysine |
| **12** | Uracil | Riboflavin | Nicotinic acid | Choline | Inositol | Valine |

Source: Holliday (1956)


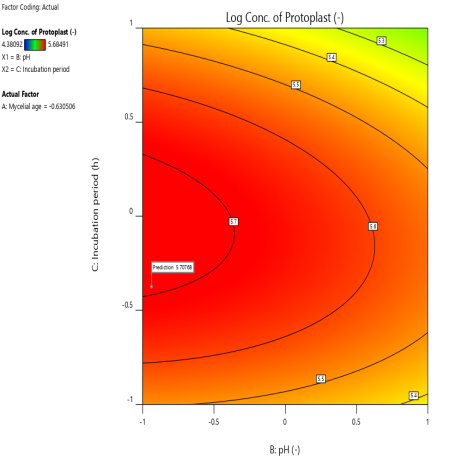

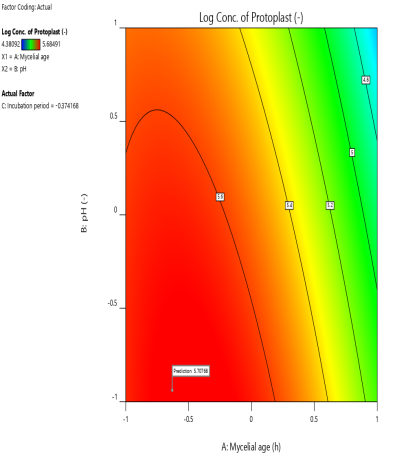

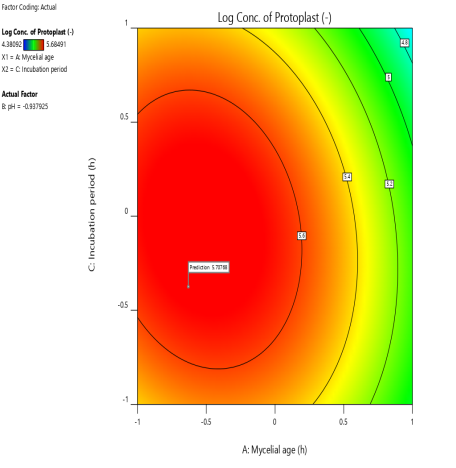


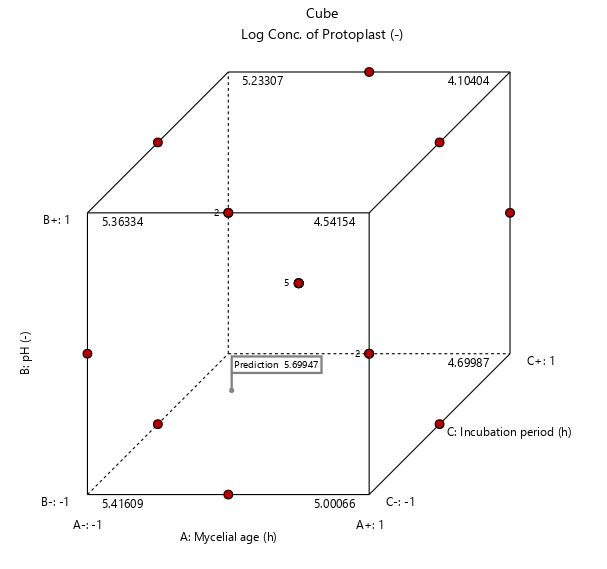

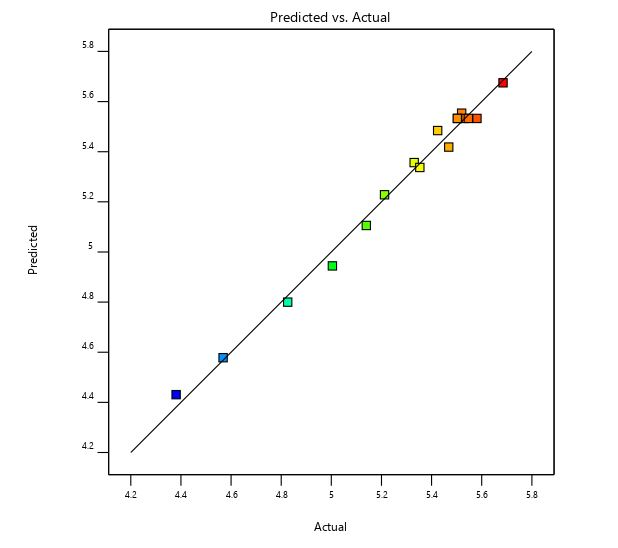


Fig. S1 Box-Behnken designed (BBD) response surface optimization (RSM) of conditions for maximum isolation of protoplasts

**Table S2 Protoplast fusant L-asparaginase screening**

| S/N | Fusant code | Mean L-ASNase activity ± standard deviation (U) |
| --- | --- | --- |
| 1 | Fusant-01 | 9823.48 ± 892.41 |
| 2 | Fusant-02 | 7462.48 ± 671.25 |
| 3 | Fusant-03 | 20892.11 ± 2452.48 |
| 4 | Fusant-04 | 10904.87 ± 3362.48 |
| 5 | Fusant-05 | 6294.90 ± 425.91 |
| 6 | Fusant-06 | 24564.74 ± 4093.32 |
| 7 | Fusant-07 | 4261.58 ± 827.67 |
| 8 | Fusant-08 | 8472.47 ± 435.22 |
| 9 | Fusant-09 | 15726.46 ± 3242.47 |
| 10 | Fusant-10 | 19843.27 ± 4416.34 |
| 11 | Fusant-11 | 21736.49 ± 4156.23 |
| 12 | Fusant-12 | 10493.26 ± 2416.88 |
| 13 | Fusant-13 | 13293.49 ± 3167.51 |


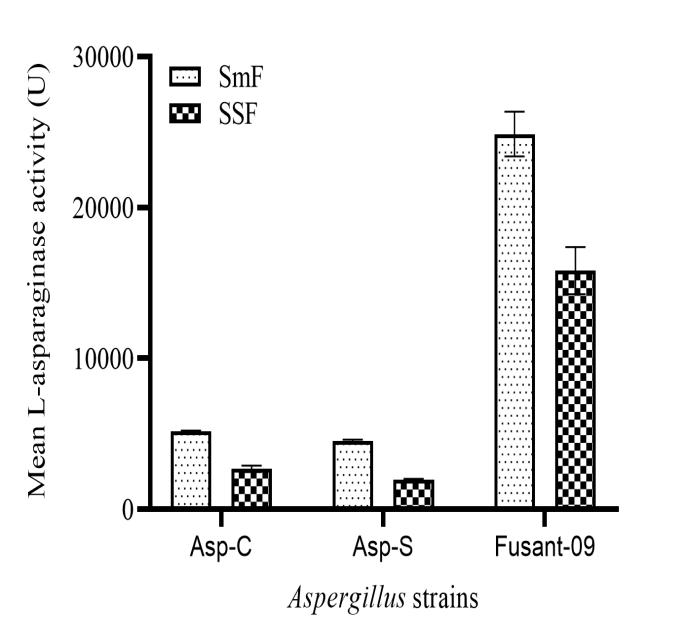

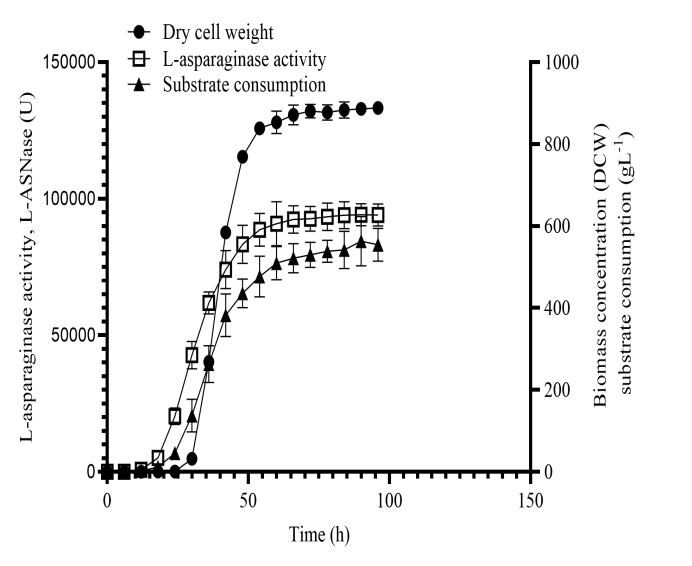


**A**

**B**


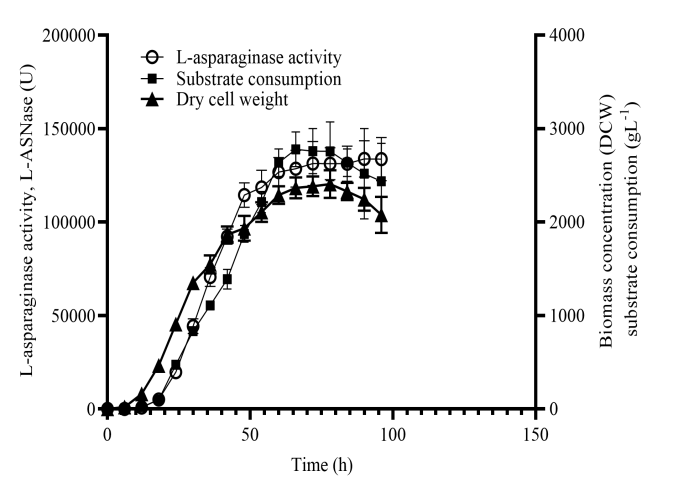

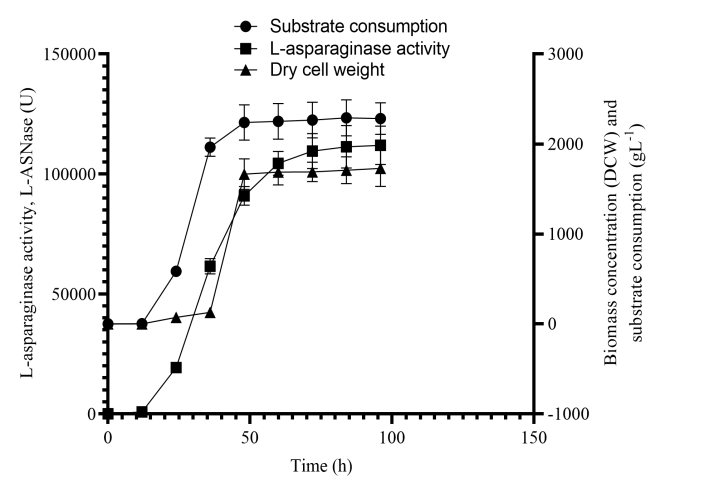


**D**

**C**

Fig. S2 Comparative evaluation of L-asparaginase production by fusant-09, Asp-C (*Aspergillus candidus*) and Asp-S (*Aspergillus sydowii*) in (**A**) submerged (SmF) and solid-state fermentation (SSF); (**B**) batch kinetics; (**C**) fed-batch kinetics (**D**) continuous fermentation kinetics


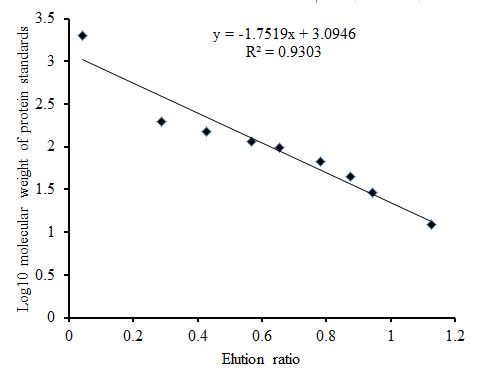


Fig. S3 Determination of Fusant-06 L-ASNase molecular weight by plotting log molecular weight of standard proteins against elution ratio and solving the regression equation using the elution ratio of the test L-ASNase (0.587)

**>L-asparaginase [*Aspergillus candidus* strain UCCM 00116]**

MAKTQDHVFTDRGGVIENRHLVHAAIVDAQGKLLYSVGDPSRITLVRSAAKPAQALAVLETGAPKQFGFD

DADLALMCASHNGEARHISRAFAMLAKVDAREQDLRCGGHAALSASVNRAWIKSDYTPTEICNNCSGKHV

GMLGGSKAIGAAIADYHLPTHPIQLRVKRVVEDLCGLEADSCQWGIDGCNLPAPAFPLHYLGKMYAALSA

AADSMAVDCSASARERGLSRIYHAMTQYPELVGGEGRFCTALMQAFGGSLVGKVGADGCYGIGIRASEAT

DRVGAAGAIGIAVKIEDGNLEILYAAVMEILEQLQIGTRDARGRLADFHRPVITNSAGVVTGHTSHEVIV

RPAMALAGKHERTTYSILSLDFGSVFSL (378 aa, Molecular weight = 72.64 kDa)

**>L-asparaginase [*Aspergillus sydowii* strain UCCM 00124]**

MTIKPWLLSALGHSGSASPDLYPRATNETYVFTEANGLNFTAFSYSLPNVTIFATGGTIAGSDYSSWATT

GYTSWAVHKLWLIDSVPSMLDVANYAGVQDANWGSEDITSDILIYMSKEINRDVCDDPTMAGTVIDHGTD

TKTNANTMDTPYAMEMGYLCELIEENLKFFYPPVKPTGKVWFDITNVTEIPRYDILFYYEDMHNDTLYNA

KSRILWGDVFDLGTD (225 aa, Molecular weight = 44.36 kDa)

**>L-asparaginase [*Aspergillus* interspecies fusant-09]**

MHYTAGFRDSERSIARKSHSTSYGANSMPKLELEIKYDGRHSGKELDGEFSTNDHSGQKDHDGKWLSKHGHGFDFQGFDGSHELSKDHSWNMDNSKFLEMSGEWEYRTEYGHWSGMNSWNSGDFERSMSGEKSLWKLAHSFAHDGESGEHDRTYRSAHWGEGSYDHSNFWENETFNYYHETSHENMDNGSYSQSYRTEHEYDGQTEYDDTDRGDWEFKSMSRDFEGDTRYSWSNDMSGDMSKSLSPSPWMSREPSLSHSLWDFKSLDMSHSRQWLSLDPSHSWSMYTEAGSAGSFWGDFQGSRWTRSTWREFYGEQERTEGHWEYGERERRQFSDWNSMWKMNLMAFEMAYHEYWKDGEMRNSDQMSNKWNSKHKSMSLDAGHILLKAQNWTERASSGAHAGEERSDAYFSDACKSVCVSLAMWNWTEHAGEEKWHEPILEYWKWLSHEGSHAWSGHELMSNDLEPSKSLAPLEMWHEGEHSTEYAWQWETWHDGQSGWRSMALWLRKLYMAL (513 aa, Molecular weight = 116.4 kDa)

Fig. S4 Amino acid profiles of the L-asparaginases from study *Aspergillus* strains


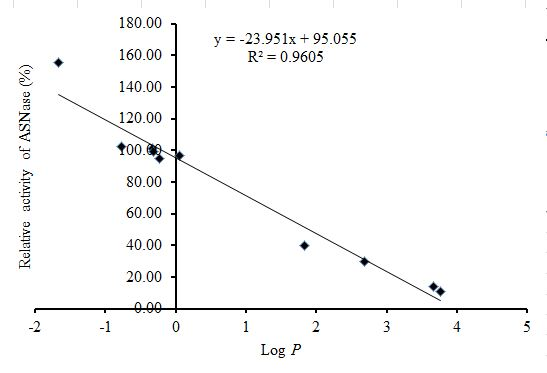


Fig. S5 Relationship between octanol-water partition coefficients of organic solvents and relative ASNase activity


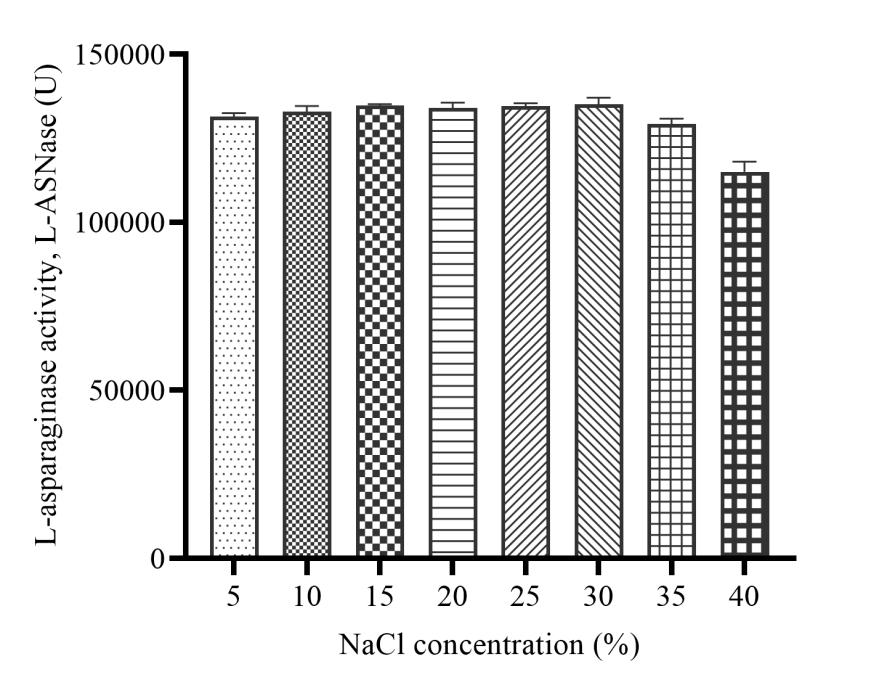


Fig. S6 Effect of NaCl on L-asparaginase activity
